# Characterizing and Comparing Adverse Drug Events Documented in 2 Spontaneous Reporting Systems in the Lower Mainland of British Columbia, Canada: Retrospective Observational Study

**DOI:** 10.2196/52495

**Published:** 2024-01-18

**Authors:** Erica Y Lau, Amber Cragg, Serena S Small, Katherine Butcher, Corinne M Hohl

**Affiliations:** 1 Department of Emergency Medicine University of British Columbia Vancouver, BC Canada; 2 Centre for Clinical Epidemiology and Evaluation Vancouver Coastal Health Research Centre Vancouver, BC Canada; 3 Pharmaceutical Science Vancouver General Hospital Vancouver Coastal Health Vancouver, BC Canada

**Keywords:** adverse drug event reporting systems, side effect, side effects, drug, drugs, pharmacy, pharmacology, pharmacotherapy, pharmaceutic, pharmaceutics, pharmaceuticals, pharmaceutical, medication, medications, patient safety, health information technology, pharmacovigilance, adverse, safety, HIT, information system, information systems, reporting, descriptive statistics, monitoring

## Abstract

**Background:**

Robust adverse drug event (ADE) reporting systems are crucial to monitor and identify drug safety signals, but the quantity and type of ADEs captured may vary by system characteristics.

**Objective:**

We compared ADEs reported in 2 different reporting systems in the same jurisdictions, the Patient Safety and Learning System–Adverse Drug Reaction (PSLS-ADR) and ActionADE, to understand report variation.

**Methods:**

This retrospective observational study analyzed reports entered into PSLS-ADR and ActionADE systems between December 1, 2019, and December 31, 2022. We conducted a comprehensive analysis including all events from both reporting systems to examine coverage and usage and understand the types of events captured in both systems. We calculated descriptive statistics for reporting facility type, patient demographics, serious events, and most reported drugs. We conducted a subanalysis focused on adverse drug reactions to enable direct comparisons between systems in terms of the volume and events reported. We stratified results by reporting system.

**Results:**

We performed the comprehensive analysis on 3248 ADE reports, of which 12.4% (375/3035) were reported in PSLS-ADR and 87.6% (2660/3035) were reported in ActionADE. Distribution of all events and serious events varied slightly between the 2 systems. Iohexol, gadobutrol, and empagliflozin were the most common culprit drugs (173/375, 46.2%) in PSLS-ADR, while hydrochlorothiazide, apixaban, and ramipril (308/2660, 11.6%) were common in ActionADE. We included 2728 reports in the subanalysis of adverse drug reactions, of which 12.9% (353/2728) were reported in PSLS-ADR and 86.4% (2357/2728) were reported in ActionADE. ActionADE captured 4- to 6-fold more comparable events than PSLS-ADR over this study’s period.

**Conclusions:**

User-friendly and robust reporting systems are vital for pharmacovigilance and patient safety. This study highlights substantial differences in ADE data that were generated by different reporting systems. Understanding system factors that lead to varying reporting patterns can enhance ADE monitoring and should be taken into account when evaluating drug safety signals.

## Introduction

Over 2 million Canadians visit an emergency department every year because of an adverse drug event (ADE), an unintended and harmful event related to medication use [1,2]. ADEs incur over 700,000 hospital admissions, and cost over CAD $1 billion (USD $7.48 million) in annual health care expenditures across Canada [2,3]. The importance of addressing this issue cannot be overstated: the World Health Organization (WHO) has identified the prevention of ADEs as an urgent global public health priority [4].

In response to this pressing concern, Canada implemented regulations outlined in the Protecting Canadians from Unsafe Drugs Act (Vanessa’s Law) which came into full effect on December 16, 2019. This federal legislation mandates prompt reporting of serious adverse drug reactions (ADRs; a subtype of ADEs) and medical device incidents from hospitals to Health Canada within 30 days of documentation [5]. These regulations serve as a safeguard to protect patients and improve drug surveillance.

Postmarketing pharmacovigilance is crucial in the detection, assessment, and prevention of ADEs under real-world conditions [6,7]. Among the various methods used, spontaneous reporting stands out as one of the most widely adopted approaches in pharmacovigilance [8]. When patients or health professionals spontaneously report ADEs, drug safety monitoring agencies evaluate and integrate these reports into databases, enabling ongoing identification of safety signals [7,8]. This method of surveillance captures data from a broad population and allows us to detect drug safety signals that may not have been identified in the randomized trials used for drug licensing and monitor rare ADEs to medications [9].

It is important to recognize, however, that there is considerable variation in ADE reporting systems worldwide in terms of their design, data fields, terminologies [10], and implementations, which may impact the volume and type of ADEs reported [11]. Variation in design also leads to a lack of standardization of reports, which can in turn prohibit interoperability or effective exchange of ADE reports between systems and may prevent comparisons of ADE events, rates, and risk factors across systems [10].

Despite this variation, the diversity of systems may also be a strength. Each system has the potential to complement others, enhancing the overall quantity and quality of ADE data, if variation in design leads to variation in reporting behaviors or the types of reports that can be entered [12]. To leverage this untapped potential, we need to better understand and compare the events collected through diverse reporting systems [13]. Understanding similarities and differences between systems will enable researchers and drug safety monitoring agencies to more effectively use existing data for accurate signal detection, especially for new or rare ADEs, and prioritize the investigation of drug safety signals. This knowledge will also aid stakeholders in optimizing the design and implementation of new reporting systems to enhance ADE data collection and drug safety surveillance and better align systems with their intended purpose [10].

Health Canada, the regulatory authority for postmarketing pharmacovigilance in Canada, oversees the Canada Vigilance Program, collecting reports of suspected ADEs since 1965. Health professionals and consumers can voluntarily submit reports through various channels, including a web-based platform, phone, fax, or mail. Hospitals are required to submit written reports within 30 days, and Health Canada allows them flexibility by permitting the use of existing systems and processes to meet reporting requirements. With Health Canada’s approval, hospitals may use a third party, such as a regional health authority or other reporting programs, to submit reports [14].

The province of British Columbia (BC) currently uses 2 approved spontaneous reporting systems that enable hospitals to comply with Vanessa’s Law mandates: the BC Patient Safety and Learning System–Adverse Drug Reaction (PSLS-ADR) reporting form and ActionADE. Briefly, PSLS-ADR was developed and implemented as the first province-wide, web-based platform and supports hospitals in meeting the mandatory reporting requirements [14]. ActionADE, implemented later in the timeline, is a research-driven, web-based app that aims to prevent unintentional redispensation of harmful medications by facilitating the sharing of ADE information between providers across health care settings. ADE reporting occurs as a byproduct of enabling safer care provision (Multimedia Appendix 1) [15].

These 2 systems enable a comparison of the quality and quantity of ADE data generated using 2 different designs. Our objective is to describe and compare the ADEs that health care providers documented using PSLS-ADR and ActionADE during the first 3 years following the implementation of Vanessa’s Law.

## Methods

### ADE Reporting Systems

#### About PSLS-ADR

BC Patient Safety and Learning System (PSLS) is an initiative of the BC Patient Safety Task Force, developed in collaboration with all 6 provincial health authorities and the Health Care Protection Program, which is part of the Risk Management Branch of the Ministry of Finance that insures BC hospitals [16]. BC PSLS is a web-based safety event reporting and management information system designed to support the identification, investigation, and analysis of safety and risk-related events, including safety hazards, near misses, and adverse events [17]. The system underwent a pilot phase in 2007 and was subsequently implemented province-wide in 2008. BC PSLS has been instrumental in promoting patient safety within the health care system in BC [16].

In response to the introduction of Vanessa’s Law and in collaboration with Health Canada, BC PSLS launched PSLS-ADR as a new add-on to the existing system in 2014 and released an updated version in 2019 [18,19]. PSLS-ADR is accessible to health care facilities in all health authorities across BC, including acute care hospitals, long-term care facilities, and outpatient clinics. Authorized health care professionals with access to the secure health authority network, including employees, medical staff, paramedics, contractors, students, and volunteers, can submit reports to PSLS-ADR [15]. Once a report is submitted, the system notifies the medication safety officer in the respective health authority to review and respond to the event [20]. The health authorities send eligible reports to Health Canada for Vanessa’s Law reporting requirements. Reports are not made available to care providers and not integrated into the electronic medical record. They are only generated for the purposes of pharmacosurveillance (Multimedia Appendix 2).

The PSLS-ADR data fields are based largely on the Canada Vigilance Adverse Reaction Reporting Form, with additional questions enabling medication safety officers, pharmacy representatives, and others to follow-up with reporters or patients, if necessary [20]. The PSLS-ADR reporting form contains 26 required data fields that collect information about the patient, the adverse reaction (eg, seriousness), the suspected health products (types, name, route used, therapy dates, and treatments), and the reporters.

#### About ActionADE

Previous studies found that 32.5% of ADE cases observed in emergency departments are repeat events [21], often occurring due to the unintentional represcription or redispensation of the same or a same-class medication as one that previously caused harm [22]. This recurrence is attributed to the lack of effective means to communicate and integrate ADE information into clinical workflows. ActionADE, a research-driven initiative, was developed to address this communication gap [23,24].

In collaboration with the Ministry of Health, Vancouver Coastal Health, a technology partner, and health professional organizations and clinicians, our research team developed and piloted ActionADE between 2016 and 2019 using participatory design principles and data standards that were evaluated and subsequently pilot tested to optimize the system’s usability [10,11,15,24-28]. In 2020, we began the implementation of ActionADE in 1 hospital (Vancouver General Hospital) and then expanded its use to 6 hospitals operated by Vancouver Coastal Health and Providence Health Care as part of a research initiative. Although providers were encouraged to use ActionADE, they maintain complete autonomy in choosing between the PSLS-ADR and ActionADE systems to meet their needs.

ActionADE is a web-based app that allows providers to document and communicate ADE information, bidirectionally through its integration (or linkage) with BC’s central drug database (PharmaNet). ActionADE was accessible to a subset of care providers with an eligible prescriber identification number issued by their respective regulatory college (ie, physicians, pharmacists, and nurse practitioners) [29]. Eligible clinicians submit reports to ActionADE from a designated health authority network, and the data are shared with clinicians within the patient’s circle of care via PharmaNet and used to create safety alerts when community pharmacists attempt to redispense culprit or same-class medications. ActionADE complements the PSLS-ADR system by automating ADE reporting to Health Canada (Multimedia Appendix 3).

The ActionADE data fields were developed based on a systematic review of ADE reporting systems worldwide and participatory action research with clinician end users and are compatible with Health Canada’s Canada Vigilance Adverse Reaction Reporting Form [10,11,15,27,30]. As ActionADE is integrated with PharmaNet, several fields auto-populate based on the patient’s personal health number, including patient’s personal and demographic information (ie, name, date of birth, and sex), reporter’s information (ie, name, role, and site), and patient’s 14-month medication dispensation history. To create a new report, the system auto-populates the patient’s information and medication dispensation history, as well as the reporter’s information. ActionADE contains 5 required data fields that collect information about the suspect drugs, which is auto generated based on the medication dispensation history or added manually, the ADE type, and details of the event (eg, symptoms or diagnosis, outcome, and certainty; Multimedia Appendix 4).

### Study Design

In this retrospective observational study, we analyzed reports documented in PSLS-ADR and ActionADE entered by providers at health care facilities operated by the Vancouver Coastal Health Authority (excluding Providence Health Care, as PSLS-ADR data were unavailable from those facilities) in BC, Canada, between December 1, 2019, to December 31, 2022.

For PSLS-ADR, we included reports documented by authorized health care professionals (eg, employees, medical staff, paramedics, contractors, students, and volunteers) from >120 health care facilities across the province, including hospital, urgent and primary care, long-term care facilities, and community health centers’ clinics. For ActionADE, we included reports documented by eligible clinicians from 4 hospitals where ActionADE is implemented: Lions Gate Hospital, Richmond Hospital, UBC (University of British Columbia) Hospital, and Vancouver General Hospital (Multimedia Appendix 5) [31].

We divided this study’s period into 4 phases: baseline period, when all hospitals across BC only used PSLS-ADR (December 2019 to February 2020); year 1 (March 2020 to November 2020), when 1 hospital (Vancouver General Hospital) had the option to use ActionADE for piloting purposes while all other sites in BC exclusively used PSLS-ADR; and year 2 (December 2020 to November 2021) and year 3 (December 2021 to December 2022), when the 4 hospitals had the option to use PSLS-ADR or ActionADE and all other sites in BC exclusively used PSLS-ADR (Multimedia Appendix 6).

### Data Sources

For this study, we requested ADE reports from PSLS-ADR from the BC PSLS central office and retrieved reports documented in ActionADE during the same period from the ActionADE database. We obtained information about hospital characteristics through the Information Access and Privacy Services at Provincial Health Services Authority, including number of beds, population served, and the number of emergency department visits per year.

### Data Extraction

To allow for direct comparisons between the 2 systems, we combined similar variables wherever possible. A clinical pharmacist classified all free-text drug entries from the PSLS-ADR reports into the equivalent generic drug name that would be present if the same report were entered into ActionADE based on the provincial formulary. We translated continuous age from ActionADE into the age categories in PSLS-ADR. We combined information across platforms to produce combined variables for report date, patient demographics (age group and sex), types of ADE (ADRs and nonadverse drug reactions), ADE outcomes (death, emergency visit, hospitalized or hospital extended, life threatening, worsened preexisting condition, permanent disability and fetal defect, other, and unknown) [27], and reporter information (role and facility).

### Statistical Analysis

#### Comprehensive Analysis

First, we performed a comprehensive statistical analysis to provide a global view of coverage, usage, and the types of information captured by both reporting systems. We included all events from both reporting systems, excluding reports related to user errors (eg, duplicate reports), refuted allergies, and reports with incomplete data. We calculated descriptive statistics (eg, means and SDs or frequency and percentages) for the following variables: total number and types of the reporting facilities (hospital vs nonhospital), patient’s age group, patient’s sex, roles of reporters, proportion of serious events, and the 10 most reported culprit drugs for all events and serious events. We defined serious events based on the Health Canada’s definition. This definition includes ADEs that require in-patient hospitalization or prolongation of existing hospitalization, cause congenital malformation, result in persistent or significant disability or incapacity, are life-threatening, or result in death [14].

#### ADR Analysis

To allow for direct comparisons between the 2 systems, we then conducted a subsample analysis that only included ADR reports (a subtype of ADE) that met Health Canada’s definition and that could have been reported in both systems. According to Health Canada, ADRs encompass harmful and unintended responses to a health product, including any undesirable patient effects suspected to be associated with health product use. This definition includes unintended effects, health product abuse, overdoses, interactions (including drug-drug and drug-food interactions), and unusual lack of therapeutic efficacy, all of which are considered reportable adverse reactions [14,32,33]. We included eligible ADR reports from both reporting systems from sites where both systems were available, excluding reports related to user errors (eg, duplicate reports), refuted allergies, and reports with incomplete data. We calculated descriptive statistics for the following variables: patient’s age group, patient’s sex, proportion of serious events, the 10 most reported culprit drugs, and mean monthly counts of all events and serious events during each phase of this study period, stratified by reporting system. We conducted all analyses using SAS statistical software (version 9.4; SAS Institute).

### Ethical Considerations

The UBC (University of British Columbia) clinical research ethics board approved of this research (H18-01332 and H22-00312) and provided a waiver for obtaining informed consent as this study meets the Tri-Council Policy Statement minimal risk criteria.

## Results

### Comprehensive Analysis

**Figure 1:**
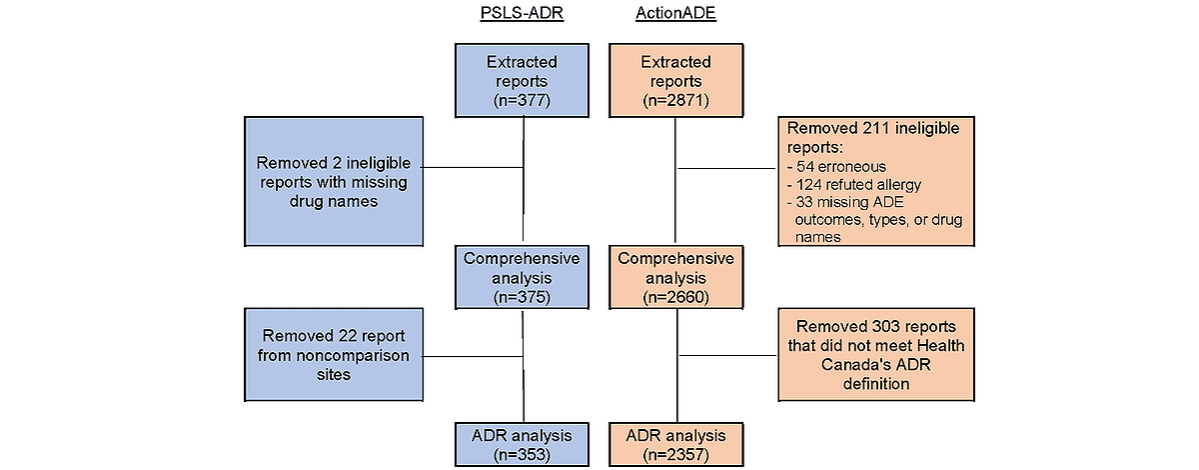
Flow diagram. ADE: adverse drug event; ADR: adverse drug reaction; PSLS-ADR: Patient Safety and Learning System–Adverse Drug Reaction.

We extracted 3248 reports from both reporting systems. After removing 213 reports related to refuted allergies, erroneous reports, and reports with incomplete data, the analytic cohort for the comprehensive analysis comprised 3035 unique ADEs reported in either system (Figure 1). Of these, 12.4% (375/3035) were entered in PSLS-ADR and 87.6% (2660/3035) were reported in ActionADE.

Approximately 50% of the events occurred in male patients in both PSLS-ADR (178/375) and ActionADE (1285/3035). The highest proportion of events were from patients aged 45-64 years (32.8%, 123/375) in PSLS-ADR and aged 75-84 years (25.3%, 674/2660) in ActionADE. In total, 12 facilities (5 hospitals and 7 nonhospital facilities) entered reports in PSLS-ADR. The primary reporters in PSLS-ADR were medical imaging staff or technicians (170/375, 45.3%) and pharmacists (174/375, 46.4%). Of the 4 hospitals that entered reports in ActionADE, pharmacists were the reporter for 92.1% (2451/3035) of the events. The proportion of serious events was 36% (135/377) in PSLS-ADR and 28.2% (749/3035) in ActionADE (Table 1).

In PSLS, the most common culprit drugs were iohexol, gadobutrol, and empagliflozin, accounting for 46.2% (173/375) of all events. Empagliflozin, ibuprofen, and iohexol represented 11.8% (16/135) of serious events (Tables 2 and 3). Iohexol and gadobutrol are both contrast agents used for diagnostic imaging, whereas empagliflozin is an oral medication primarily prescribed for managing type 2 diabetes mellitus and ibuprofen is an oral, over-the-counter nonsteroidal anti-inflammatory drug used to relieve pain, reduce inflammation, and alleviate fever.

In ActionADE, the most common culprit drugs were hydrochlorothiazide, ramipril, and apixaban, which accounted for 10.5% (356/3391) of all events; hydrochlorothiazide, empagliflozin, and apixaban represented 11.1% (105/951) of serious events (Tables 2 and 3). Hydrochlorothiazide and ramipril are commonly prescribed for hypertension. Apixaban, an oral anticoagulant, is primarily used for stroke prevention in patients with atrial fibrillation and for treatment and prevention of venous thromboembolism.

**Table 1:** Descriptive statistics of all events included in the comprehensive analysis by reporting system^a^.

Characteristics			PSLS-ADR^b^ (n=375), n (%)		ActionADE^c^ (n=2660), n (%)
**Type of reporting facilitates**					
	Hospitals	5 (41.6)		4 (100)	
	Nonhospitals	7 (58.4)		0 (0)	
**Patient age group (y)**					
	<1-19	11 (2.9)		22 (0.8)	
	20-44	83 (22.1)		299 (11.2)	
	45-64	123 (32.8)		523 (19.7)	
	65-74	67 (17.9)		544 (20.5)	
	75-84	52 (13.9)		674 (25.3)	
	>84	39 (10.4)		598 (22.5)	
**Patient sex**					
	Male	178 (47.5)		1285 (48.3)	
**Role of reporter**					
	Physicians	Suppressed^d^		204 (7.7)	
	Nurses	27 (7.2)		—^e^	
	Medical imaging staff or technologists	170 (45.3)		—^e^	
	Nurse practitioners	Suppressed		5 (0.2)	
	Pharmacists	174 (46.4)		2451 (92.1)	
	Others	Suppressed		—^e^	
Proportion of serious events^f^			135 (36.0)		749 (28.2)

^a^The comprehensive analysis included all events from both reporting systems excluding reports related to errors, refuted allergy, and incomplete data on study variables.

^b^PSLS-ADR: Patient Safety and Learning System–Adverse Drug Reaction.

^c^ADE: adverse drug event.

^d^Cell sizes <5 are suppressed.

^e^These personnel are not eligible to report in ActionADE.

^f^Serious events are those with an outcome of fetal defect, permanent disability, hospitalization, extended hospitalization, life threatening, or death.

**Table 2:** Most frequently reported culprit drugs for all events in the comprehensive analysis by reporting systems.

System and drug		n (%)
**PSLS-ADR^a^ (n=375)**		
	Iohexol	154 (41.1)
	Gadobutrol	12 (3.2)
	Empagliflozin	7 (1.9)
	Rivaroxaban	7 (1.9)
	Furosemide	6 (1.6)
	Nivolumab	6 (1.6)
	Ramipril	6 (1.6)
	Unknown generic drug	6 (1.6)
	Acetylsalicylic acid	5 (1.3)
	Ibuprofen	5 (1.3)
**ActionADE^b^ (n=2660)**		
	Hydrochlorothiazide	113 (4.2)
	Apixaban	103 (3.9)
	Ramipril	92 (3.5)
	Acetylsalicylic acid	88 (3.3)
	Warfarin	88 (3.3)
	Rivaroxaban	79 (3)
	Furosemide	77 (2.9)
	Empagliflozin	63 (2.4)
	Metformin HCL^c^	52 (2)
	Spironolactone	50 (1.9)

^a^PSLS-ADR: Patient Safety and Learning System–Adverse Drug Reaction.

^b^ADE: adverse drug event.

^c^HCL: hydrochloride.

**Table 3:** Most frequently reported culprit drugs for serious events^a^ in the comprehensive analysis by reporting systems.

System and drug		n (%)
**PSLS-ADR^b^ (n=135)**		
	Empagliflozin	6 (4.4)
	Ibuprofen	5 (3.7)
	Iohexol	5 (3.7)
	Nivolumab	5 (3.7)
	Acetylsalicylic acid	Suppressed^c^
	Glyburide	Suppressed
	Rivaroxaban	Suppressed
	Allopurinol	Suppressed
	Amlodipine besylate	Suppressed
	Apixaban	Suppressed
**ActionADE^d^ (n=749)**		
	Hydrochlorothiazide	43 (5.7)
	Empagliflozin	26 (3.5)
	Apixaban	24 (3.2)
	Furosemide	20 (2.7)
	Acetylsalicylic acid	18 (2.4)
	Rivaroxaban	18 (2.4)
	Candesartan cilexetil	16 (2.1)
	Ramipril	16 (2.1)
	Chlorthalidone	16 (2.1)
	Spironolactone	15 (2)

^a^Serious events are those with an outcome of fetal defect, permanent disability, hospitalization, extended hospitalization, life threatening, or death.

^b^PSLS-ADR: Patient Safety and Learning System–Adverse Drug Reaction.

^c^Cell sizes <5 are suppressed.

^d^ADE: adverse drug event.

### ADR Analysis

We included a total of 2728 reports that met Health Canada’s definition of an ADR from facilities that had the option of using either reporting system during this study’s period (Figure 1) [32,33]. Of the included reports, 12.9% (353/2728) were entered in PSLS-ADR, while the majority (2357/2728, 86.4%) were reported in ActionADE.

The distribution of ADR reports by patient sex, age, primary reporters and proportion of serious events for both systems were similar to the comprehensive analysis (Table 4). However, each reporting system revealed distinct patterns of reporting. In PSLS-ADR, iohexol, gadobutrol, and empagliflozin accounted for 44.8% (168/353) of all events, while empagliflozin, ibuprofen, and nivolumab represented 12.1% (16/133) of serious events. In ActionADE, hydrochlorothiazide, ramipril, and apixaban accounted for 12% (284/2357) of all events. Furthermore, hydrochlorothiazide, empagliflozin, and apixaban represented 13.4% (88/671) of serious events (Tables 5 and 6).

**Figure 2:**
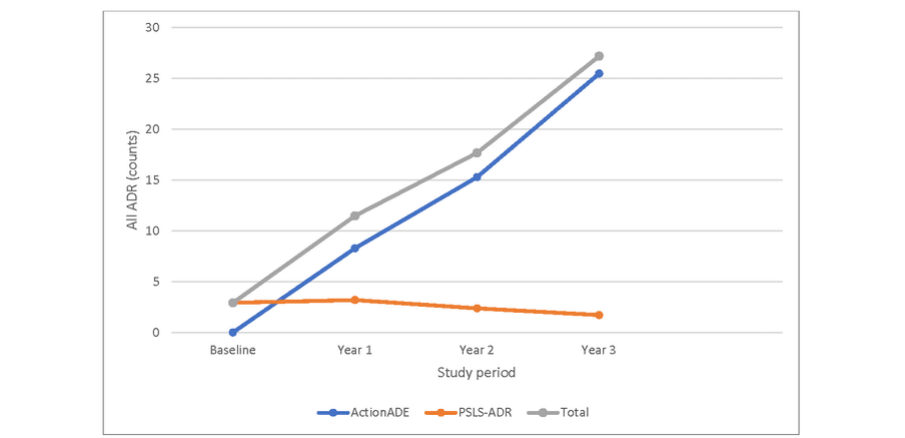
Mean monthly counts of all events meeting Health Canada’s ADR definitions across common reporting sites during this study’s period. ADR: adverse drug reaction; PSLS-ADR: Patient Safety and Learning System–Adverse Drug Reaction.

**Figure 3:**
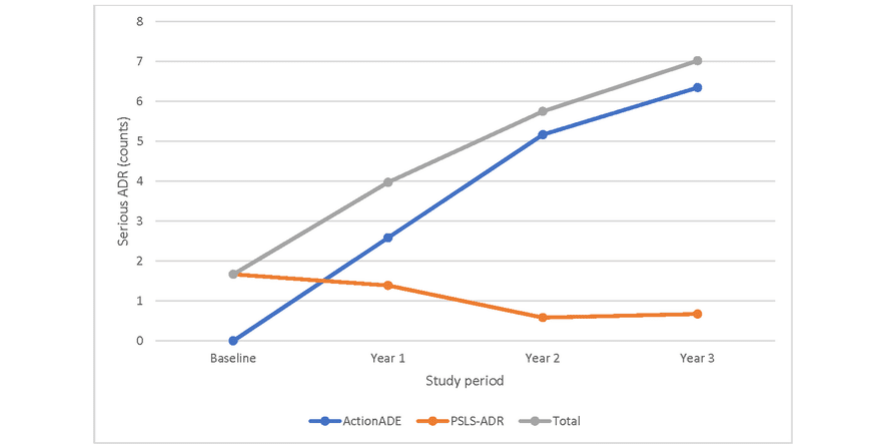
Mean monthly counts of serious events meeting Health Canada’s ADR definitions across common reporting sites during this study’s period. ADR: adverse drug reaction; PSLS-ADR: Patient Safety and Learning System–Adverse Drug Reaction.

A direct comparison in events reportable through both the PSLS-ADR and ActionADE systems revealed an increase in event reporting, including serious events, following the implementation of ActionADE (Figures 2 and 3). Baseline measurements indicate that the mean monthly counts of all events and serious events across sites were 2.9 (95% CI 2.2 to 3.6) and 1.7 (95% CI 0.8 to 2.5), respectively. In period 3, the mean monthly counts of all events and serious events across sites escalated to 27.2 (95% CI 20.4 to 34.0) and 7.0 (95% CI 4.9 to 9.2), respectively, reflecting a 9- and 4-fold increase over time. Furthermore, the mean monthly counts of all events and serious events during this study’s period within the ActionADE system were 6- and 4-fold greater than that of PSLS-ADR.

**Table 4:** Descriptive statistics of events meeting Health Canada’s ADR^a^ definition^b^ across common reporting sites^c^ by reporting system.

Characteristics		PSLS-ADR^d^ (n=353), n (%)	ActionADE^e^ (n=2357), n (%)
**Patient age group (y)**			
	<1-19	11 (3.1)	18 (0.8)
	20-44	77 (21.8)	239 (10.1)
	45-64	114 (32.3)	450 (19.1)
	65-74	64 (18.1)	494 (21)
	75-84	49 (13.9)	606 (25.7)
	>84	38 (10.8)	550 (23.3)
**Patient sex**			
	Male	173 (49)	1114 (47.3)
**Role of reporter**			
	Physicians	Suppressed^f^	161 (6.8)
	Nurses	22 (6.2)	—^g^
	Medical imaging staff or technologists^h^	155 (43.9)	—
	Nurse practitioners	Suppressed	5 (0.2)
	Pharmacists	173 (49)	2190 (92.9)
	Others^h^	Suppressed	—
Proportion of serious events^i^		133 (37.7)	671 (28.5)

^a^ADR: adverse drug reactions.

^b^According to Health Canada adverse drug reaction includes unintended effects, health product abuse, overdoses, interactions (including drug-drug and drug-food interactions), and unusual lack of therapeutic efficacy.

^c^Common reporting sites included Vancouver General, University of British Columbia, Lions Gate, and Richmond Hospitals.

^d^PSLS-ADR: Patient Safety and Learning System–Adverse Drug Reaction.

^e^ADE: adverse drug event.

^f^Cell sizes <5 are suppressed.

^g^Not available.

^h^These personnel are not eligible to report in ActionADE.

^i^Serious events are those with an outcome of fetal defect, permanent disability, hospitalization, extended hospitalization, life threatening, or death.

**Table 5:** Most frequently reported culprit drugs for all events meeting Health Canada’s ADR^a^ definitions^b^ across common reporting sites^c^ by reporting system and severity.

System and drug		n (%)
**PSLS-ADR^d^ (n=353)**		
	Iohexol	139 (39.4)
	Gadobutrol	12 (3.4)
	Empagliflozin	7 (2)
	Rivaroxaban	7 (2)
	Furosemide	6 (1.7)
	Nivolumab	6 (1.7)
	Ramipril	6 (1.7)
	Acetylsalicylic acid	5 (1.4)
	Ibuprofen	5 (1.4)
	Indapamide	5 (1.4)
**ActionADE^e^ (n=2357)**		
	Hydrochlorothiazide	109 (4.6)
	Ramipril	88 (3.7)
	Apixaban	87 (3.7)
	Acetylsalicylic acid	75 (3.2)
	Warfarin	74 (3.1)
	Rivaroxaban	71 (3)
	Empagliflozin	60 (2.5)
	Furosemide	56 (2.4)
	Metformin HCL^f^	44 (1.9)
	Ibuprofen	43 (1.8)

^a^ADR: adverse drug reaction.

^b^According to Health Canada adverse drug reaction includes unintended effects, health product abuse, overdoses, interactions (including drug-drug and drug-food interactions), and unusual lack of therapeutic efficacy.

^c^Common reporting sites included Vancouver General, University of British Columbia, Lions Gate, and Richmond Hospitals.

^d^PSLS-ADR: Patient Safety and Learning System–Adverse Drug Reaction.

^e^ADE: adverse drug event.

^f^HCL: hydrochloride.

**Table 6:** Most frequently reported culprit drugs for serious events^a^ meeting Health Canada’s ADR^b^ definitions^c^ across common reporting sites^d^ by reporting system and severity.

System and drug		n (%)
**PSLS-ADR^e^ (n=133)**		
	Empagliflozin	6 (4.5)
	Ibuprofen	5 (3.8)
	Nivolumab	5 (3.8)
	Acetylsalicylic acid	Suppressed^f^
	Glyburide	Suppressed
	Rivaroxaban	Suppressed
	Allopurinol	Suppressed
	Amlodipine besylate	Suppressed
	Apixaban	Suppressed
	Clopidogrel bisulfate	Suppressed
**ActionADE^g^ (n=671)**		
	Hydrochlorothiazide	43 (6.7)
	Empagliflozin	25 (3.7)
	Apixaban	20 (3)
	Acetylsalicylic acid	19 (2.8)
	Chlorthalidone	16 (2.4)
	Ramipril	14 (2.1)
	Rivaroxaban	14 (2.1)
	Ibuprofen	13 (1.9)
	Warfarin	13 (1.9)
	Candesartan cilexetil	12 (1.8)

^a^Common reporting sites included Vancouver General, University of British Columbia, Lions Gate, and Richmond Hospitals.

^b^ADR: adverse drug reaction.

^c^According to Health Canada adverse drug reaction includes unintended effects, health product abuse, overdoses, interactions (including drug-drug and drug-food interactions), and unusual lack of therapeutic efficacy.

^d^Serious events are those with an outcome of fetal defect, permanent disability, hospitalization, extended hospitalization, life threatening, or death.

^e^PSLS-ADR: Patient Safety and Learning System–Adverse Drug Reaction.

^f^Cell sizes <5 are suppressed.

^g^ADE: adverse drug event.

## Discussion

### Principal Findings

Our study aimed to describe and compare ADEs reported using 2 distinct reporting systems that were developed and implemented in different ways. Both PSLS-ADR and ActionADE are currently in use in BC in the first 3 years following the implementation of Vanessa’s Law. We observed differences in reports between the 2 systems regarding their coverage, usage, and the type of ADE data captured.

PSLS-ADR had broader coverage, collecting data from various health care facilities including community health centers, vaccination clinics, and outpatient clinics. Its user base was more diverse including physicians, nurses, medical imaging staff or technologists, nurse practitioners, pharmacists, and other professionals. In contrast, ActionADE coverage was limited to ADEs identified in patients presenting to 4 participating hospitals, with clinical pharmacists as its primary user. The broader coverage of PSLS-ADR can be attributed to its established position as a provincial safety event reporting platform; its accessibility to a broader range of health professions; and a federal mandate for hospitals to stimulate reporting using health authority wide communication efforts including email blasts, information on health authority websites, and presentations to provider groups. Leveraging the insights gained from PSLS-ADR, our research team is actively collaborating with key stakeholders to broaden ActionADE’s app. The Vancouver Coastal Health Authority, where ActionADE is presently in use, has recently endorsed it as a standard practice for ADE reporting in new care settings, including long-term care homes, in-patient wards, and community clinics.

Although PSLS-ADR exhibited broader coverage, ActionADE demonstrated higher usage. Our comparative analysis revealed that the average monthly counts of all events and serious events in ActionADE were 6 and 4 times higher, respectively, than in the PSLS-ADR system. Several factors might contribute to these discrepancies in reporting rates. First, PSLS-ADR was designed solely for Vanessa’s Law compliance, with reports forwarded to Health Canada for surveillance purposes. ActionADE, on the other hand, serves the dual purpose of functioning as both a clinical communication tool and a means of complying with Vanessa’s Law, thus improving patient safety [15]. Reports entered into ActionADE are used to generate preventive alerts in community pharmacies when pharmacists attempt redispensation of a drug that has previously caused the patient harm, which have demonstrated preliminary effectiveness [34]. The potential impact of reporting in ActionADE on patient safety is likely a motivating factor for providers to report ADEs [35]. Furthermore, ActionADE has a proactive implementation support mechanism, which has been shown to be instrumental in enhancing providers’ adoption of the reporting platform [35]. Finally, ActionADE used participatory design principles to optimize its design to facilitate use by end users and is integrated with PharmaNet to enable prepopulation of fields to allow reporters to generate reports ≤2 minutes, whereas PSLS-ADR users noted that reports can take 20 minutes to complete [34].

The 2 systems captured adverse events to different culprit drugs. This can be attributed to the more limited accessibility of ActionADE. The most reported drugs in PSLS-ADR were iohexol and gadobutrol, and correspondingly, medical imaging staff or technologists made up a significant proportion of reporters. This suggests that the current workflow for ADR reporting of radiopharmaceuticals is designated to medical imaging staff or technologists. Imaging staff or technologists were unable to use ActionADE at the time of this study due to PharmaNet legislation, which requires that users have prescriber ID restricting use to physicians, pharmacists, and nurse practitioners. This restriction has resulted in fewer radiopharmaceutical ADRs to be reported, as pharmacists generally do not work in radiology departments.

ActionADE frequently captured hydrochlorothiazide-related events, while only a few of such events were captured in PSLS-ADR. Among the ADRs associated with hydrochlorothiazide, electrolyte disturbances, and acute kidney injury were found to be the most common [34], involving multiple additional contributing factors. The specific functionality offered by ActionADE, such as the ability to specify the provider’s certainty that the patient’s presentation and the option to update or refute events based on new information or alternative diagnoses, likely played a role in encouraging clinicians to report these more complex events [11,15,27,30].

Ibuprofen was the second most commonly reported culprit drug related to serious events in PSLS-ADR, but it barely made the top 10 in ActionADE. This discrepancy may be due to the over-the-counter status of ibuprofen, which means patients can access the medication without a prescription and bypass communication about ADEs from ActionADE that is built into the prescription dispensation process.

While our study primarily focused on comparing these 2 systems, it is crucial to view these findings in the broader context of ADE reporting. Despite these disparities, both systems play vital roles in contributing to patient safety by capturing valuable information on ADEs. PSLS-ADR is an effective means of capturing radiopharmaceutical-related ADEs by imaging staff and technicians who are not trained in taking medication histories or ADE assessments, while ActionADE is more effective for pharmaceutical-related ADEs by clinical pharmacists that are reported and communicated on a patient-level to improve safety. These systems work in a complementary manner, catering to different areas of the health care system and capturing unique data and thus offering a more comprehensive picture of ADEs. For example, a common signal between the 2 systems might indicate a more serious issue for a specific drug irrespective of context (eg, empagliflozin). These findings suggest the need for careful attention to the design and implementation of these systems to ensure they effectively serve their intended users and context of use and ensure data resulting from each system are interpreted correctly by end users. The absence of reporting of one type of event may reflect design, implementation, or user characteristics rather than the absence of these events.

### Limitations

To our knowledge, this is the first study to directly compare 2 ADE reporting systems operating within the same jurisdiction. While the results of our study provided valuable insights into the differences between these systems, it is important to acknowledge several limitations that warrant consideration in interpreting the results. First, our study sample was confined to 2 reporting systems, which may not fully encapsulate the diversity of all systems employed across health care settings globally. As a result, the findings may not be generalizable to other reporting systems. Second, our data set was limited to facilities that used PSLS-ADR or ActionADE for reporting. This reduces the generalizability of our findings to the wider array of health care facilities in BC or nationally. It is plausible that unaccounted-for variations in data and reporting practices among facilities not deploying these 2 systems could exist. Third, our study may be susceptible to unmeasured and uncontrolled confounding variables. For example, the level of organizational emphasis on ADE reporting, differences in implementation, available resources, and providers’ perceptions could have affected the usage and coverage of the 2 systems under study. This variability might have further influenced the nature of ADE information reported. Fourth, the relatively small number of drugs resulting in ADEs prevented us from conducting a robust quantitative comparison of these events. Furthermore, the data we used were a snapshot in time and may not reflect changes in reporting systems or health care facilities that have occurred since then. Lastly, we consciously chose not to draw comparisons with other studies examining the frequently reported culprit drugs from spontaneous reporting systems in other jurisdictions. This decision stems from the recognition that the diversity in ADE reports—both in terms of numbers and types—is intricately tied to factors such as system design, geography, population characteristics, drug exposures, and the medical system itself. To facilitate meaningful comparisons across studies, a more robust surveillance system is needed.

### Conclusions

Understanding the differences between reporting systems can inform future systems design and improvement, including changes to user training and implementation, and inform the use of forthcoming data and procurement decisions for reporting systems. Further research could explore how to integrate the strengths of both systems, potentially leading to more comprehensive safety data to facilitate drug and patient safety and inform pharmacoepidemiologic studies. Continuous evaluation and improvement are essential considering the significant role these systems play to improve our health systems.

## Appendix

Multimedia Appendix 1 Characteristics of (Patient Safety and Learning System–Adverse Drug Reaction) PSLS-ADR and ActionADE.

Multimedia Appendix 2 Screenshot of Patient Safety and Learning System–Adverse Drug Reaction (PSLS-ADR).

Multimedia Appendix 3 Screenshot of ActionADE.

Multimedia Appendix 4 Data fields included in Patient Safety and Learning System–Adverse Drug Reaction (PSLS-ADR) and ActionADE.

Multimedia Appendix 5 Characteristics of sites that had options to use Patient Safety and Learning System–Adverse Drug Reaction (PSLS-ADR) or ActionADE systems.

Multimedia Appendix 6 Descriptions of the 4-phase study period.

## Abbreviations

ADE: adverse drug event
ADR: adverse drug reaction
BC: British Columbia
PSLS-ADR: Patient Safety and Learning System–Adverse Drug Reaction
PSLS: Patient Safety and Learning System
UBC: University of British Columbia
WHO: World Health Organization
