# Supplementary material for: Characterizing and Comparing Adverse Drug Events Documented in 2 Spontaneous Reporting Systems in the Lower Mainland of British Columbia, Canada: Retrospective Observational Study
Source: JMIR Hum Factors. 2024 Jan 18;11:e52495. doi: 10.2196/52495 (PMC10835584; doi:10.2196/52495)
Supplement: Multimedia Appendix 1 [file humanfactors_v11i1e52495_app1.docx]

**Multimedia Appendix 1.** Characteristics of PSLS-ADR and ActionADE.

| Aspects | PSLS-ADR | ActionADE |
| --- | --- | --- |
| What is the purpose for developing the system? | To enable hospitals to meet Vanessa’s Law mandates for reporting serious adverse drug events to Health Canada | To prevent the unintentional re-dispensation of harmful medications by facilitating the sharing of adverse drug events across healthcare settings. |
| Where is the system available? | All health authorities in British Columbia, including acute care hospitals, long-term care facilities, and outpatient clinics. | Vancouver Coastal Health Authority in British Columbia. It is primarily used within emergency departments and pharmacy departments across six hospitals. |
| Who is eligible to use the system? | All authorized healthcare professionals with access to the secure health authority network, including employees, medical staff, paramedics, contractors, students, and volunteers. | A subset of authorized healthcare professionals with access to the secure health authority network, including physicians, pharmacists, and nurse practitioners. |
| How to access the systems? | Access the system through the PSLS icon on the banner bar of the CERNER electronic medical record systems. | Access the system through the PSLS icon on the banner bar of the CERNER electronic medical record systems. Once on the PSLS landing page, select the ActionADE icon. |
| What types of ADE are being captured by the system? | Serious adverse drug events | All types of adverse drug events |
| How many required data fields  (see Appendices 2 and 3 for data field content) | 26 | 5 |
| How are the reports being used? | - Once a report is submitted, the system notifies the medication safety officer in the respective health authority to review and respond to the event. - The health authority sends eligible reports to Health Canada for Vanessa’s Law reporting requirements. | - Once a report is submitted, the system shares the data with PharmaNet (BC’s central drug database), enabling the generation of safety alerts in community pharmacies. - The health authority sends eligible reports to Health Canada for Vanessa’s Law reporting requirements. |

PSLS-ADR= Patient Safety and Learning System- Adverse Drug Reaction Form
