# Supplementary material for: Characterizing and Comparing Adverse Drug Events Documented in 2 Spontaneous Reporting Systems in the Lower Mainland of British Columbia, Canada: Retrospective Observational Study
Source: JMIR Hum Factors. 2024 Jan 18;11:e52495. doi: 10.2196/52495 (PMC10835584; doi:10.2196/52495)

**Multimedia Appendix 2.** Screenshot of PSLS-ADR.

Access PSLS-ADR from the banner bar of Cerner (hospital electronic medical records system)


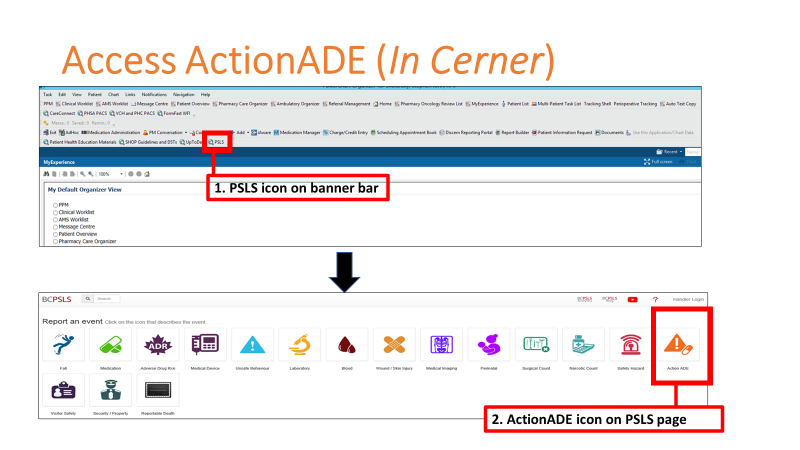


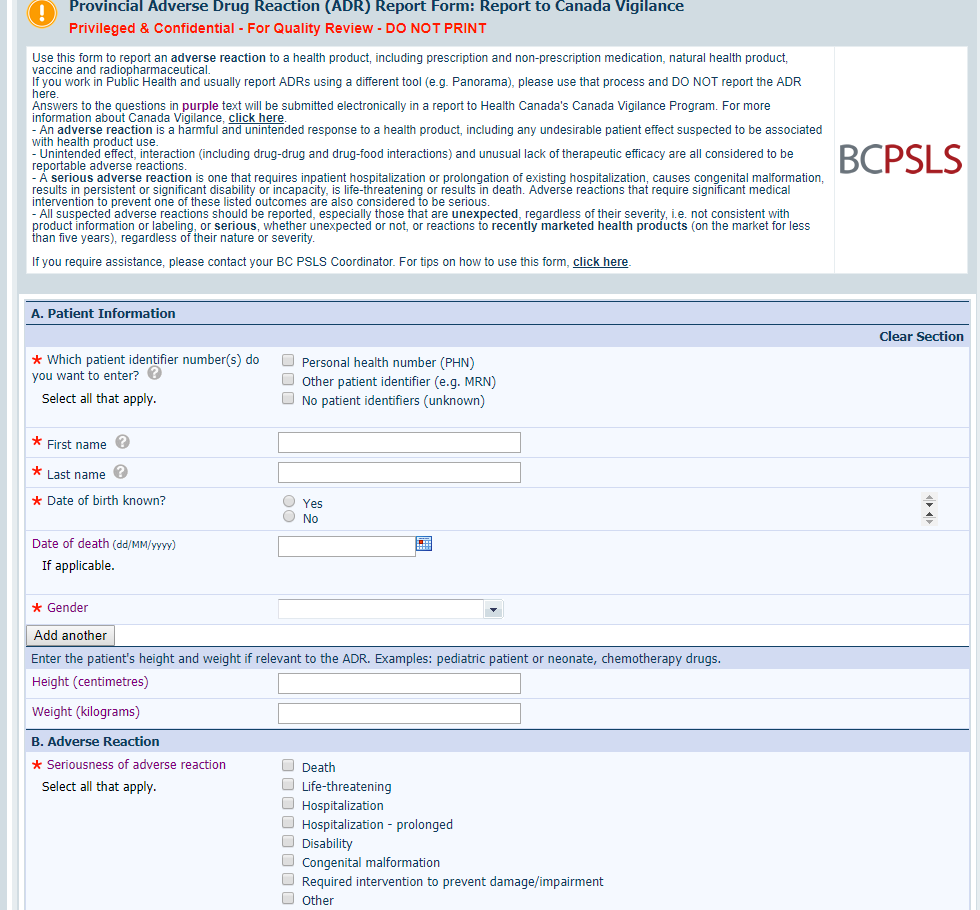


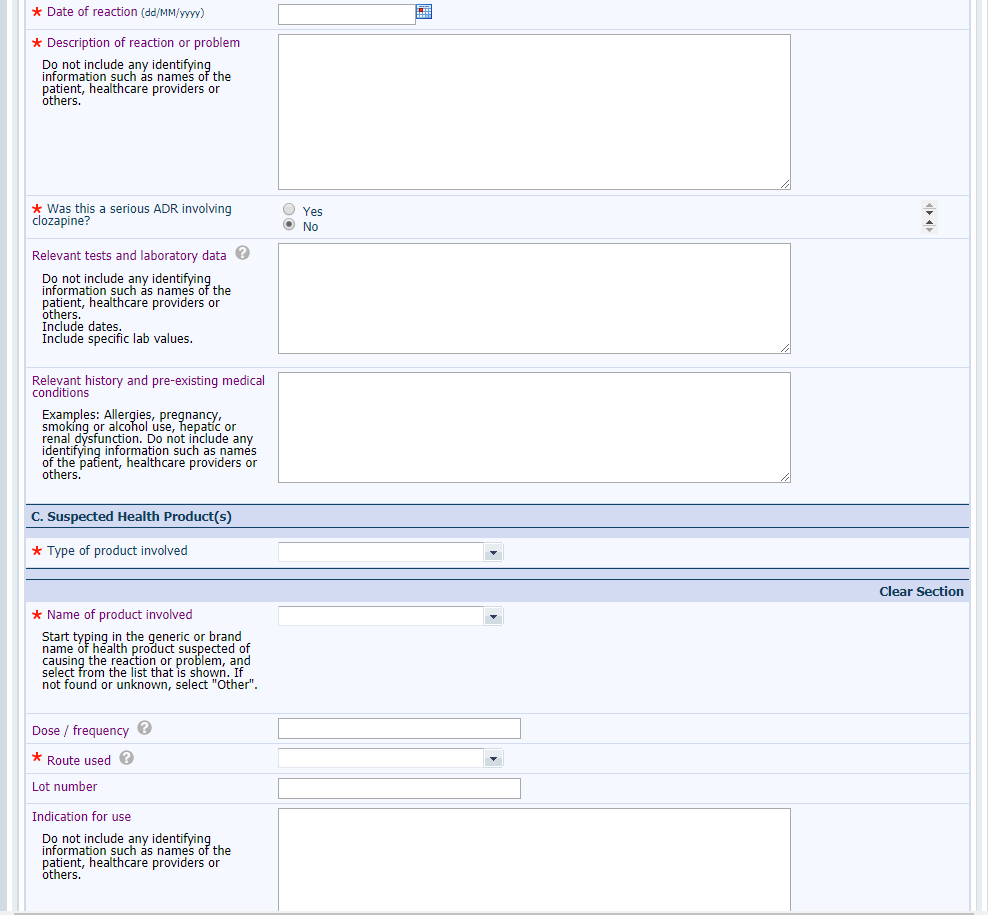

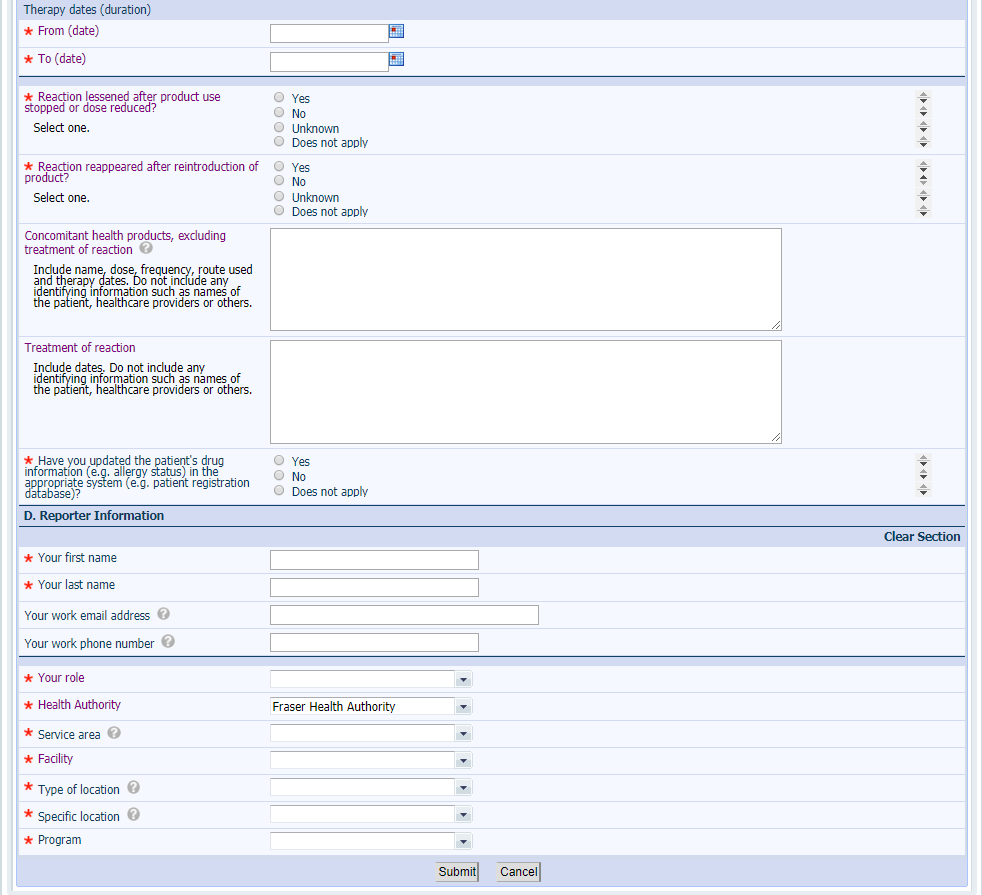

Supplement: Multimedia Appendix 2 [file humanfactors_v11i1e52495_app2.docx]
