# Supplementary material for: Characterizing and Comparing Adverse Drug Events Documented in 2 Spontaneous Reporting Systems in the Lower Mainland of British Columbia, Canada: Retrospective Observational Study
Source: JMIR Hum Factors. 2024 Jan 18;11:e52495. doi: 10.2196/52495 (PMC10835584; doi:10.2196/52495)

**Multimedia Appendix 3.** Screenshot of ActionADE.

1. Access ActionADE through the PSLS icon on banner bar of Cerner (hospital electronic medical records system). Once logged in, select ActionADE icon.


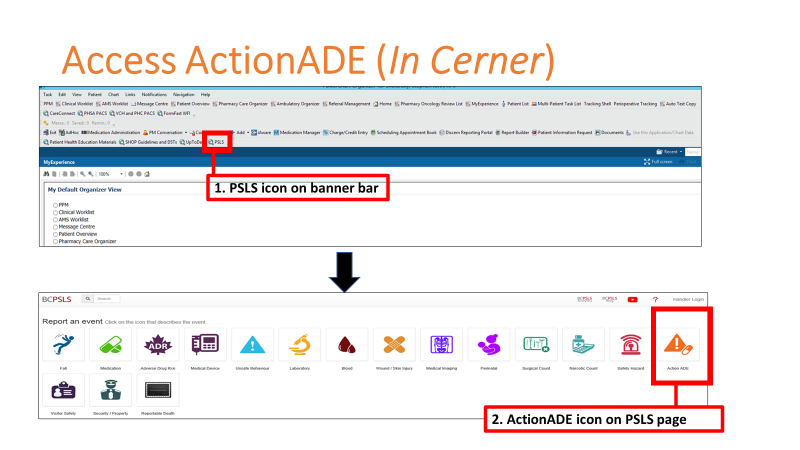


1. Once logged in, complete the following steps to add a new adverse drug event report.


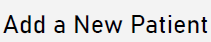


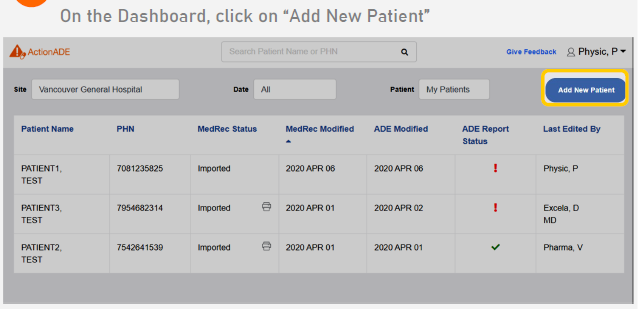


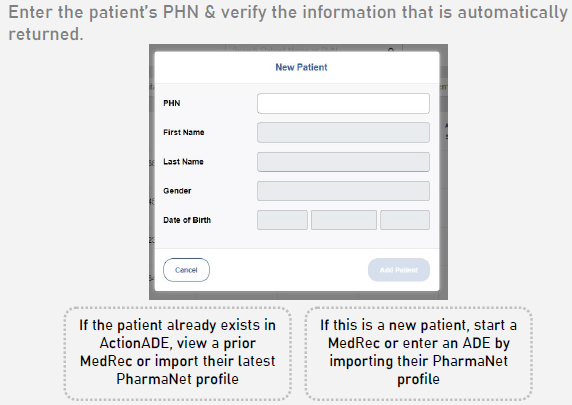


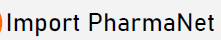


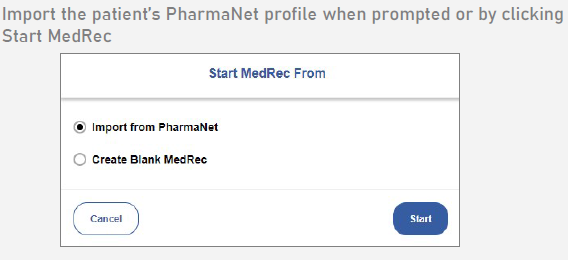


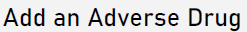


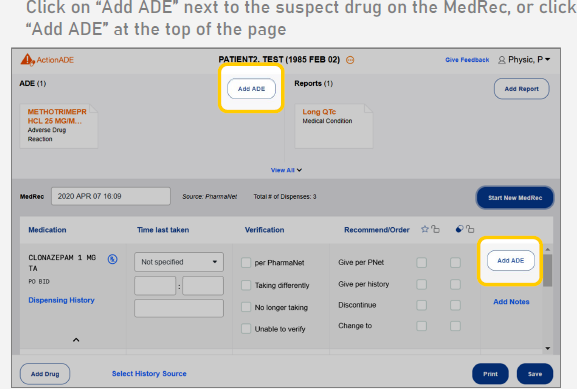


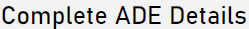

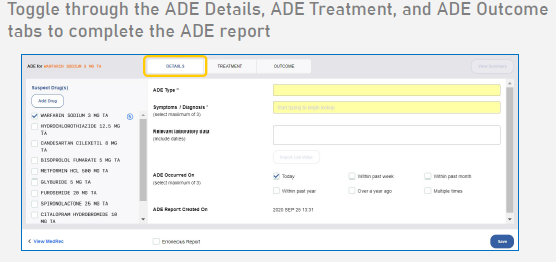


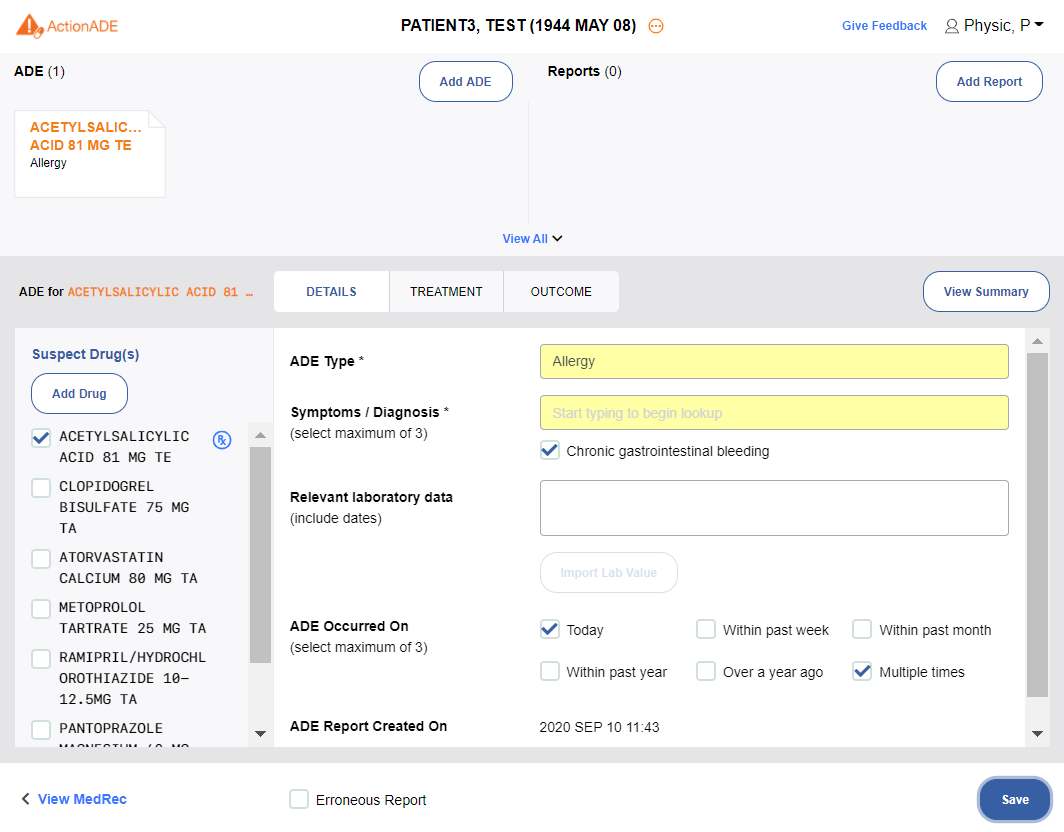


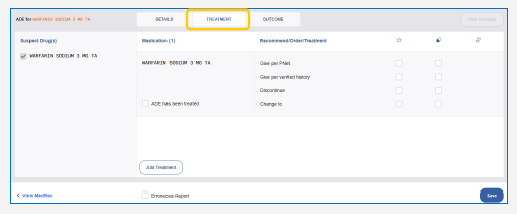


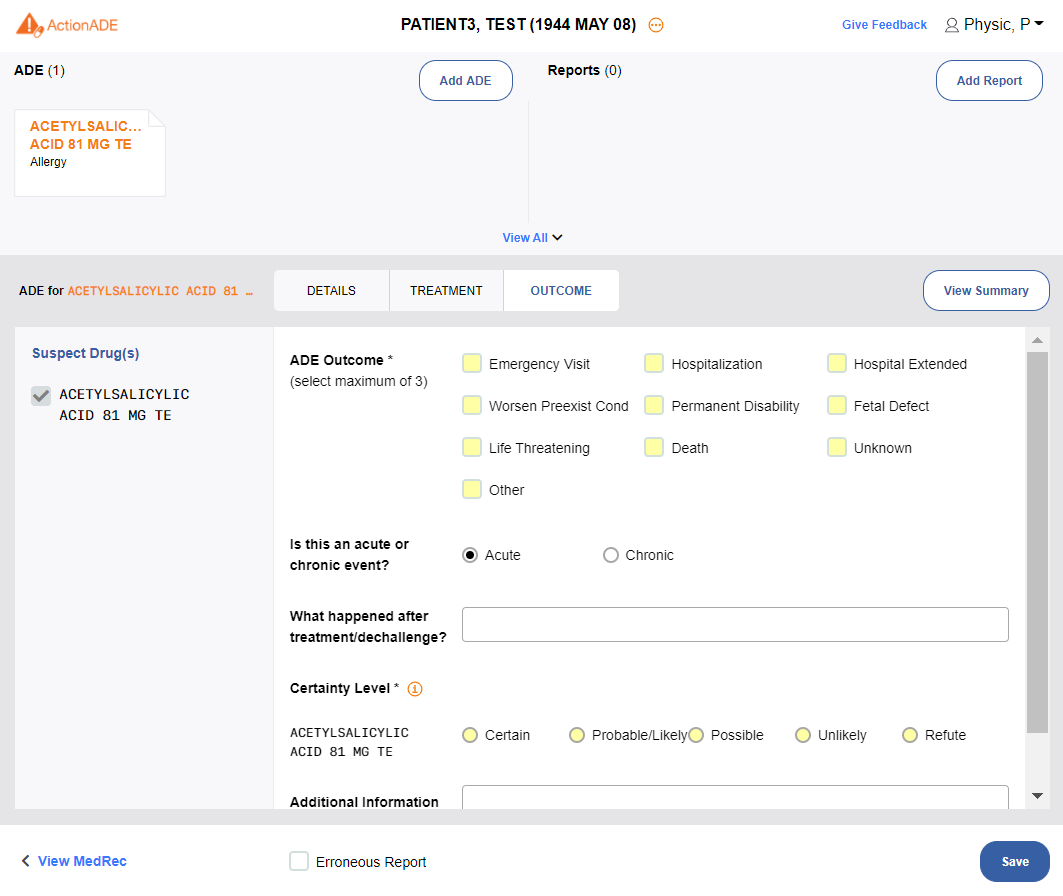

Supplement: Multimedia Appendix 3 [file humanfactors_v11i1e52495_app3.docx]
