# Supplementary material for: Characterizing and Comparing Adverse Drug Events Documented in 2 Spontaneous Reporting Systems in the Lower Mainland of British Columbia, Canada: Retrospective Observational Study
Source: JMIR Hum Factors. 2024 Jan 18;11:e52495. doi: 10.2196/52495 (PMC10835584; doi:10.2196/52495)
Supplement: Multimedia Appendix 4 [file humanfactors_v11i1e52495_app4.docx]

**Multimedia Appendix 4.** Data fields included in PSLS-ADR and ActionADE.

| PSLS-ADR | ActionADE |
| --- | --- |
| Patient information |  |
| *Which patient identifier number(s) do you want to enter?   - Personal Health Number (PHN) - Other (e.g., MRN) - No patient identifiers | *Personal Health Number (PHN) |
| *First name | ^First name |
| *Last name | ^Last name |
| *Date of birth known.   - Yes - No | ^Date of birth |
| Date of birth |  |
| Date of death (if applicable) |  |
| *Gender   - Female - Male - Transgender - Undetermined or other - Unknown | ^Gender   - Female - Male |
| Enter the patient’s height and weight if relevant to the ADR:   - Height - Weight |  |
| Adverse drug event details |  |
| *Seriousness of adverse reaction:   - Death - Life-threatening - Hospitalization - Hospitalization – prolonged - Disability - Congenital malformation - Required intervention to prevent damage / impairment - Other | *ADE Outcome:   - Death - Life threatening - Hospitalization - Hospital extended. - Permanent disability - Fetal defect - Other - Unknown - Worsen pre-existing condition - Emergency visit |
| *Date of reaction | Date of report OR “ADE Occurred on” |
| - *Description of reaction or problem | *ADE Type   - Adverse drug reaction - Allergy - Incorrect Drug - Subtherapeutic dose - Supratherapeutic dose - Treatment failure - Drug withdrawal, - Drug interaction - Non-adherence - Other |
|  | *Symptom / diagnosis   - MedDRA preferred terms. User types first three letters of symptom/diagnosis for results list to appear. User can enter up to 3 symptoms and diagnoses, populating a visible symptom/diagnosis list |
| Was this a serious ADR involving clozapine?   - Yes - No |  |
| Relevant tests and laboratory data   - Free text | Relevant laboratory data (include dates)   - Free text |
| Relevant history or pre-existing medical conditions | *Additional information* |
| Suspected health product (s) |  |
| *Type of product involved:   - Medication or IV fluid - Biological product (e.g., vaccine) - Contrast media - Radiopharmaceutical | - |
| *Name of product involved   - Name of product involved is selected from a drop-down list | *Drug Identification Number (DIN) |
| Dose / frequency   - free text | Dose taken   - free text |
|  | Dose unit   - g - mg - mcg - mL - mEq - mmol - mcL - mol - mcmol |
| *Route used | Route   - Oral - SC - IM - IV - Topical - Inhale - Sublingual |
|  | Frequency   - Once - Daily - Nightly - Bedtime - twice daily - three times a day four times a day every hour - every other day - three times weekly |
| Lot number   - free text | - |
| Indication for use   - free text | Indication for drug   - MedDRA preferred terms. User types first three letters of symptom/diagnosis for results list to appear. User can enter up to 3 symptoms and diagnoses, populating a visible symptom/diagnosis list |
| *Therapy dates (duration)   - From (date) - To (date) |  |
| *Reaction lessened after product use stopped or dose reduced?   - Yes - No - Unknown - Does not apply | *What happened after treatment / dechallenge?*   - Complete resolution - Improvement without complete resolution - No change - Worsening - Unknown |
|  | *Certainty level   - Certain - Probably/Likely - Possible - Unlikely - Refute |
| *Reaction reappeared after reintroduction of product?   - Yes - No - Unknown - Does not apply |  |
| Concomitant health products, excluding treatment of reaction | *PharmaNet list* |
| Treatment of reaction | Treatment page |
| *Have you updated the patient’s drug information (e.g., allergy status) in the appropriate system (e.g., patient registration database)?   - Yes - No - Does not apply | - Give per PNet - Give per verifirf hidotry - Discontinued - Change to |
| Reporter information |  |
| * Your first name | First name |
| * Your last name | Last name |
| Your work email address | Username |
| Your work phone number | Phone number |
| *Your role   - Contracted services - Employee - Nurse - Paramedic - Physician / midwife - Student - Therapist - Other | Role   - Pharmacist - Physician - Nurse Practitioner |
| Your specific role | - |
| *Health authority   - VCH - FH - VIHA | *VCH* |
| *Service Area   - Acute - Home health - Mental health and substance use - Palliative care - Primary care - Etc. | *Acute* |
| *Facility | Hospital site   - VGH - SPH - LGH - RH - MSJ - UBCH |
| *Type of location *(many possible selections – including:*   - Contracted services - Critical care - Emergency - Fleet Operations – EHS - Fraser Cariboo – EHS - Inpatient ward / resident unit - Pharmacy - ETC. | Acute (default) |
| *Specific location: (*many possible selections)* | - |
| *Program: *(many possible selections)* | - |

PSLS-ADR= Patient Safety and Learning System- Adverse Drug Reaction Form

* denotes required data fields

^ denotes data field auto populated from PharmaNet (BC’s central drug database)
