# Supplementary material for: Characterizing and Comparing Adverse Drug Events Documented in 2 Spontaneous Reporting Systems in the Lower Mainland of British Columbia, Canada: Retrospective Observational Study
Source: JMIR Hum Factors. 2024 Jan 18;11:e52495. doi: 10.2196/52495 (PMC10835584; doi:10.2196/52495)
Supplement: Multimedia Appendix 5 [file humanfactors_v11i1e52495_app5.docx]

**Multimedia Appendix 5.** Characteristics of sites that had options to use PSLS-ADR and/or ActionADE systems.

| Site | # of beds and types | Population served | # of emergency department visits/year |
| --- | --- | --- | --- |
| Lions Gate Hospital (LGH) | 235 beds, acute care, community hospital | Urban and rural | 62,477 |
| Richmond Hospital (RH) | 434 beds, acute care, teaching hospital | Urban | 54,156 |
| Vancouver General Hospital (VGH) | 666 beds, acute care, teaching hospital | Urban | 78,993 |
| University of British Columbia Hospital (UBCH) | 87 beds, acute care, community hospital | Urban | 28,577 |

PSLS-ADR= Patient Safety and Learning System- Adverse Drug Reaction Form
