# Supplementary material for: Characterizing and Comparing Adverse Drug Events Documented in 2 Spontaneous Reporting Systems in the Lower Mainland of British Columbia, Canada: Retrospective Observational Study
Source: JMIR Hum Factors. 2024 Jan 18;11:e52495. doi: 10.2196/52495 (PMC10835584; doi:10.2196/52495)
Supplement: Multimedia Appendix 6 [file humanfactors_v11i1e52495_app6.docx]

**Multimedia Appendix 6.** Descriptions of the four-phase study period.


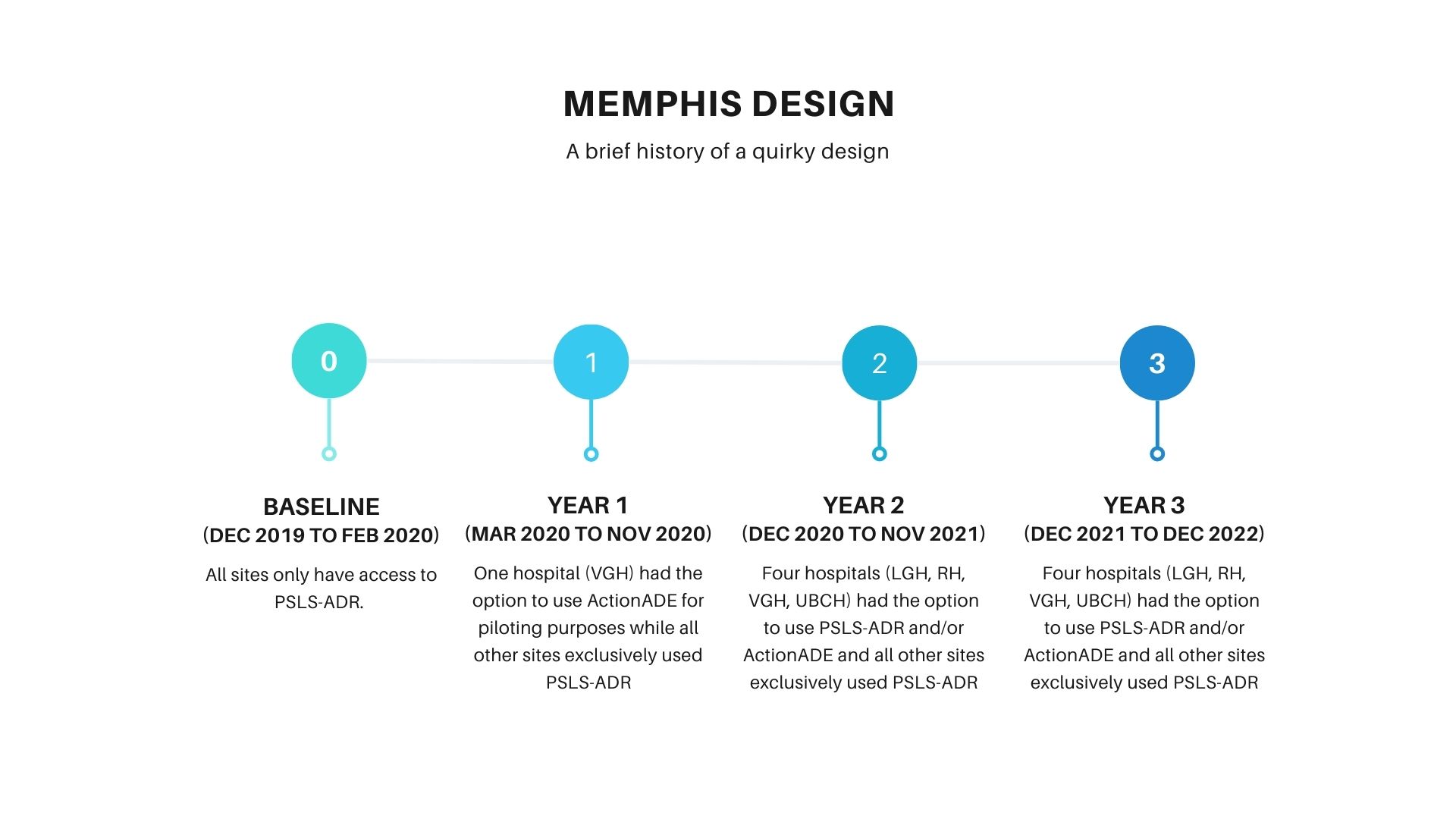


PSLS-ADR= Patient Safety and Learning System- Adverse Drug Reaction Form; LGH= Lions Gate’s Hospital, RH= Richmond Hospital; VGH, Vancouver General Hospital; UBCH= University of British Columbia Hospital
